# An antibody to IL-1 receptor 7 protects mice from LPS-induced tissue and systemic inflammation

**DOI:** 10.3389/fimmu.2024.1427100

**Published:** 2024-06-25

**Authors:** Liqiong Jiang, Lars P. Lunding, William S. Webber, Karsten Beckmann, Tania Azam, Jesper Falkesgaard Højen, Jesus Amo-Aparicio, Alberto Dinarello, Tom T. Nguyen, Ulrich Pessara, Daniel Parera, David J. Orlicky, Stephan Fischer, Michael Wegmann, Charles A. Dinarello, Suzhao Li

**Affiliations:** ^1^ Department of Medicine, University of Colorado Denver, Aurora, CO, United States; ^2^ Division of Lung Immunology, Priority Area of Chronic Lung Diseases, Research Center Borstel-Leibniz Lung Center, Borstel, Germany; ^3^ Airway Research Center North, Member of the German Center for Lung Research (DZL), Giessen, Germany; ^4^ MAB Discovery GmbH, Polling, Germany; ^5^ Department of Infectious Diseases, Aarhus University Hospital, Aarhus, Denmark; ^6^ Mucosal Inflammation Program and Division of Gastroenterology, Hepatology, and Nutrition, Department of Pediatrics, Children’s Hospital Colorado, University of Colorado, Aurora, CO, United States; ^7^ IcanoMAB GmbH, Polling, Germany; ^8^ Department of Pathology, University of Colorado Denver Anschutz Medical Campus, Aurora, CO, United States

**Keywords:** IL-1 receptor 7 (IL-1R7), interleukin-18 (IL-18), blockade, mouse, inflammation, IFNg, macrophage activation syndrome (MAS), therapeutic

## Abstract

**Introduction:**

Interleukin-18 (IL-18), a pro-inflammatory cytokine belonging to the IL-1 Family, is a key mediator ofautoinflammatory diseases associated with the development of macrophage activation syndrome (MAS).High levels of IL-18 correlate with MAS and COVID-19 severity and mortality, particularly in COVID-19patients with MAS. As an inflammation inducer, IL-18 binds its receptor IL-1 Receptor 5 (IL-1R5), leadingto the recruitment of the co-receptor, IL-1 Receptor 7 (IL-1R7). This heterotrimeric complex subsequentlyinitiates downstream signaling, resulting in local and systemic inflammation.

**Methods:**

We reported earlier the development of a novel humanized monoclonal anti-human IL-1R7 antibody whichspecifically blocks the activity of human IL-18 and its inflammatory signaling in human cell and wholeblood cultures. In the current study, we further explored the strategy of blocking IL-1R7 inhyperinflammation in vivo using animal models.

**Results:**

We first identified an anti-mouse IL-1R7 antibody that significantly suppressed mouse IL-18 andlipopolysaccharide (LPS)-induced IFNg production in mouse splenocyte and peritoneal cell cultures. Whenapplied in vivo, the antibody reduced Propionibacterium acnes and LPS-induced liver injury and protectedmice from tissue and systemic hyperinflammation. Importantly, anti-IL-1R7 significantly inhibited plasma,liver cell and spleen cell IFNg production. Also, anti-IL-1R7 downregulated plasma TNFa, IL-6, IL-1b,MIP-2 production and the production of the liver enzyme ALT. In parallel, anti-IL-1R7 suppressed LPSinducedinflammatory cell infiltration in lungs and inhibited the subsequent IFNg production andinflammation in mice when assessed using an acute lung injury model.

**Discussion:**

Altogether, our data suggest that blocking IL-1R7 represents a potential therapeutic strategy to specificallymodulate IL-18-mediated hyperinflammation, warranting further investigation of its clinical application intreating IL-18-mediated diseases, including MAS and COVID-19.

## Introduction

Discovered as an IFNγ-inducing factor, interleukin-18 (IL-18) belongs to the IL-1 family of cytokines (1–3). Similar to IL-1β, IL-18 is first synthesized as an intracellular inactive precursor and released as an active (mature) cytokine by caspase-1 cleavage (4, 5). IL-1 Receptor 5 (IL-1R5, also called IL-18 receptor alpha chain) is the ligand binding chain for mature IL-18, although this binding is of low affinity. In cells that express the co-receptor, termed IL-1 Receptor 7 (IL-1R7, also known as IL-18 receptor beta chain), IL-18 forms a high affinity complex with IL-1R5 and IL-1R7 to initiate the subsequent downstream inflammatory signaling (1, 6). IL-18 binding protein (IL-18BP), a natural inhibitor for IL-18, keeps the activity of IL-18 at bay in healthy conditions by providing a competing high affinity binding site for IL-18 (7).

In pathological conditions like macrophage activation syndrome (MAS), COVID-19 and inflammatory bowel diseases (IBD), IL-18 is upregulated and plays an important role in the disease development (1, 5, 8–13). There has been emerging interest to develop inhibitors for IL-18 to treat IL-18-mediated hyperinflammation and diseases. Former studies have examined the concepts of blocking IL-18 with the natural inhibitor IL-18BP, or antibodies against its receptor IL-1R5. However, because of the high affinity of IL-18BP for IL-18, IL-18BP also binds IL-37, an anti-inflammatory cytokine whose tertiary structure is closely related to IL-18 (1, 14, 15). Thus, use of IL-18BP to block the activity of IL-18 has the disadvantage of binding to IL-37 and reducing the anti-inflammatory function of IL-37 in disease. In fact, several studies have reported inflammatory diseases associated with low IL-37 (16–18), whereas the anti-inflammatory properties of IL-18BP are lost at high doses (19). IL-1R5, the receptor and ligand-binding chain for IL-18, also serves as a receptor for IL-37 (5, 20, 21). Hence, antibodies against IL-1R5 would concurrently block endogenous IL-37 and its anti-inflammatory functions. Indeed, some data has revealed that blocking IL-1R5 with antibodies or using IL-18BP exacerbates inflammation (22, 23). Therefore, it is important to develop alternative strategies in IL-18 blockage.

In an earlier study (6), we explored the strategy of targeting IL-18 signaling with antibodies against IL-1R7 (anti-IL-1R7), the sole accessory chain for IL-1R5 and co-receptor for IL-18 signaling (24). We found that a humanized monoclonal anti-IL-1R7 antibody to human IL-1R7 (anti-hIL-1R7) suppressed IL-18-mediated pro-inflammatory signaling and subsequent cytokine production in primary human cell cultures (6). To further investigate the application of anti-IL-1R7 in clinical trials to treat diseases associated with IL-18-mediated hyperinflammation, it is essential to evaluate the concept *in vivo* using mouse models. In the present study, we screened antibodies to mouse IL-1R7 and developed a monoclonal antibody to mouse IL-1R7 (anti-mIL-1R7) for *in vivo* mouse models with hyperinflammation. We found that the anti-mIL-1R7 inhibited IL-18-induced IFNγ production in mouse spleen and peritoneal cells and protected mice from *Propionibacterium acnes* (*P. acnes*) and lipopolysaccharide (LPS)-induced liver injury and systemic inflammation. In addition, the anti-mIL-1R7 attenuated LPS-induced acute lung inflammation in mice. Together, data from both human cell studies (6) and *in vivo* mouse model studies demonstrate that blocking IL-1R7 could be a promising therapeutic strategy to specifically modulate IL-18 signaling and IL-18-related inflammatory diseases. This warrants further investigation of the clinical potential of anti-IL-1R7 for treating patients with MAS, MAS-like clinical manifestations of COVID-19, and other IL-18-mediated inflammatory diseases.

## Results

### Identification of an effective anti-mouse IL-1R7 antibody

**Figure 1:**
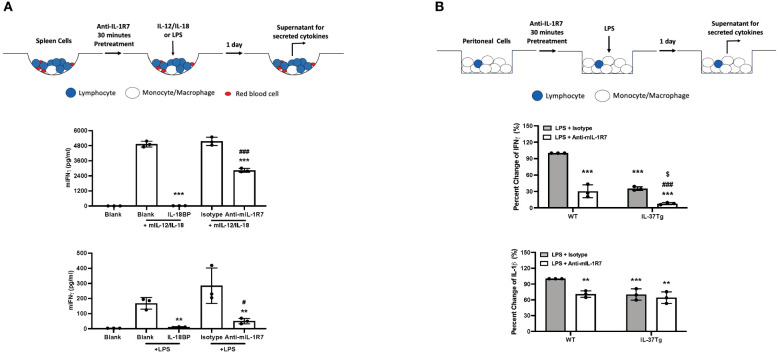
Anti-mIL-1R7 monoclonal antibody inhibits IL-18-mediated IFNγ production without affecting the anti-inflammatory function of IL-37. **(A)** Anti-mIL-1R7 monoclonal antibody down-regulates IL-12/IL-18- or LPS- stimulated IFNγ production in mouse splenocyte cultures. Primary mouse splenocytes were pre-treated with or without 5μg/mL anti-mIL-1R7 or its isotype control or 1μg/mL IL-18BP for 30 minutes before IL-12/IL-18- or LPS stimulation (as depicted in the procedure diagram). Please see *Methods* section for details. Mean ± SD of IFNγ production in the cells. N=3 for all conditions. **p < 0.01, ***p < 0.001 compared with IL-12/IL-18 or LPS alone-treated cells. #p < 0.05, ###p < 0.001 compared with the isotype control-pretreated cells. **(B)** Effects of the anti-mIL-1R7 on the anti-inflammatory function of IL-37 in LPS-stimulated mouse peritoneal cells. Thioglycolate-elicited peritoneal cells from WT or IL-37-Tg mice were pre-treated with or without anti-mIL-1R7 before LPS treatment (as depicted in the procedure diagram). Mean ± SD Percent change of LPS-induced cytokine production (LPS-induced cytokine production in WT cells was set as 100%). N=3 for all conditions. **p < 0.01, ***p < 0.001 compared with isotype-pretreated WT cells. ###p < 0.001 compared with isotype-pretreated cells from IL-37-Tg mice. $ p < 0.05 compared with anti-mIL-1R7-pretreated WT cells.

Previously, we generated an anti-hIL-1R7 antibody specific for human IL-1R7 with promising therapeutic potential in clinical studies (6). This antibody does not cross-react with mouse IL-1R7 (mIL-1R7) and thus does not affect mouse IL-12/IL-18 (mIL-12/IL-18)- or LPS- induced IFNγ in mouse splenocyte cultures (**Supplementary Figure 1**). IL-12 increases the expression of IL-1R5 and IL-1R7 and plays a synergistic role in IL-18-induced IFNγ production in cells (6, 25–27). Therefore, we used the combination of IL-12/IL-18 for IFNγ production which is directly imposed by IL-18, and LPS for IFNγ production that is mediated by IL-18 (6, 28, 29). To identify potent anti-mIL-1R7 antibodies for mouse model studies, we screened a large number of antibody clones for mouse IL-1R7-binding and mouse IL-18 (mIL-18)-blocking functions. Several potential candidates were identified (**Supplementary Figure 2**). We tested the binding of the antibody candidates to recombinant mIL-1R7 in an ELISA-based assay *in vitro* (**Supplementary Figure 2A**) and to cell surface-expressed mIL-1R7 using mIL-1R7-transfected HEK293 cells (**Supplementary Figure 2B**). As shown in the Figures, the candidates bound mIL-1R7 effectively in both assays. These antibodies were mouse IgG2a and expressed with the LALA sequence to prevent the triggering of FcγRs (6, 30, 31). These antibodies were developed to target mIL-1R7 and thus to inhibit assembling of the mouse IL-18/IL-1R5/IL-1R7 ternary complex and the subsequent pro-inflammatory signaling of mIL-18. Therefore, we first assessed the effects of the antibody candidates on mIL-12/IL-18-induced IFNγ release in mouse splenocyte cultures. As expected, the antibody candidates effectively inhibited mIL-12/IL-18-induced IFNγ (**Supplementary Figure 2C** Left). Since anti-hIL-1R7 antibodies were found to work effectively to inhibit LPS-induced IFNγ in human cell cultures (6), we also characterized the function of the anti-mIL-1R7 antibody candidates on LPS-induced IFNγ release in mouse splenocytes. As depicted in **Supplementary Figure 2C** Right, one of the antibody candidates (Batch B) showed a consistent inhibition on mIL-12/IL-18- or LPS- induced IFNγ. Based on its mIL-1R7 binding efficacy (**Supplementary Figures 2A, B** Middle) and the significant suppression on mIL-12/IL-18- or LPS- induced IFNγ release (**Supplementary Figure 2C**), the Batch B was selected as the optimal candidate for expansion. It was further investigated as the anti-mIL-1R7 antibody (anti-mIL-1R7), in comparison to a mouse IgG2a isotype control antibody (Isotype as the abbreviation) for the following *in vitro* and *in vivo* studies. The efficacy of the purified anti-mIL-1R7 on mIL-12/IL-18- or LPS- induced IFNγ release was compared to IL-18BP in mouse splenocyte cultures (**Figure 1A**). Though its suppression on IL-12/IL-18-induced IFNγ was less potent than IL-18BP, anti-mIL-1R7 inhibited LPS-induced IFNγ robustly with an efficacy comparable to IL-18BP.

### Effects of anti-mIL-1R7 on IL-37-mediated anti-inflammatory function

We next assessed the effects of anti-mIL-1R7 on inflammatory responses in thioglycolate-elicited mouse peritoneal cells from wildtype (WT) C57BL/6j mice. As shown in **Figure 1B**, anti-mIL-1R7 pretreatment significantly suppressed LPS-induced IFNγ from these peritoneal cells compared to the isotype control-pretreated cells (~ 70% reduction). Anti-mIL-1R7 also reduced LPS-induced IL-1β release by around 40% (**Figure 1B**) and TNFα by around 20% (**Supplementary Figure 3**). We next compared LPS-induced inflammatory responses between cells from WT mice and transgenic mice overexpressing human IL-37 (IL-37-Tg). As reported earlier (32), cells from IL-37-Tg mice are more resistant to LPS-induced inflammation than cells from WT mice (**Figure 1B**). IL-37 overexpression exerted similar magnitudes of inhibition on LPS-induced IFNγ and IL-1β in anti-mIL-1R7- or its isotype control- pretreated cells from the IL-37-Tg mice (**Figure 1B**), demonstrating that anti-mIL-1R7 has no interference on the anti-inflammatory function of IL-37 in cells.

### Anti-mIL-1R7 protects mice from *P. acnes* and LPS-induced systemic inflammation

**Figure 2:**
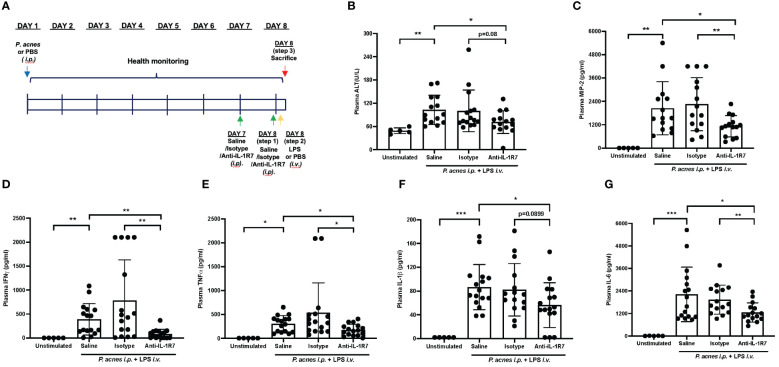
Anti-IL-1R7 pretreatment protects mice from *P. acnes*/LPS-induced systemic hyperinflammation. **(A)** Procedure diagram for the *P. acnes*/LPS model. **(B)** Plasma ALT levels, **(C)** Plasma MIP-2 levels, **(D)** Plasma IFNγ levels, **(E)** Plasma TNFα levels, **(F)** Plasma IL-1β levels, **(G)** Plasma IL-6 levels of the mice. Mean ± SD of plasma cytokines in mice challenged with *P. acnes*/LPS in the presence of anti-mIL-1R7 (anti-IL-1R7 group) or its isotype control (isotype group), or saline (saline group) for pretreatment, or in mice challenged with PBS and pretreated with saline (Unstimulated group). N ranges between 5 to 16. ∗∗∗p < 0.001, ∗∗p < 0.01, and ∗p < 0.05 for comparisons as indicated.

IL-18 was initially identified as an IFNγ-inducing factor and purified and cloned from the livers of mice treated with the bacterium *P. acnes* and subsequently challenged with LPS to induce inflammatory responses and toxic shock (2, 33). Therefore, we utilized this classic *P. acnes* and LPS treatment model (*P. acnes*/LPS) to assess the *in vivo* effects of anti-mIL-1R7 on IL-18-mediated inflammation and liver injury. Mice were challenged with *P. acnes*/LPS with or without the pretreatment of anti-mIL-1R7 (anti-IL-1R7 group), its anti-mouse IgG isotype control (isotype group) or saline (saline group) (**Figure 2A**). As shown in **Figures 2B, C**, *P. acnes*/LPS-treatment induced high levels of plasma ALT and MIP2 in mice whereas anti-IL-1R7-pretreatment down-regulated plasma ALT and MIP2 levels in comparison to saline or isotype control-pretreated groups. This is consistent with earlier reports on the correlation of ALT and MIP2 with the severity of liver damage (34, 35). We further assessed the plasma levels of proinflammatory cytokines IFNγ, TNFα, IL-1β and IL-6. As shown in **Figure 2D**, anti-IL-1R7 pretreatment markedly suppressed plasma IFNγ in comparison to pretreatments with saline or isotype control antibody. In addition, plasma TNFα, IL-1β and IL-6 were also lower in anti-IL-1R7-pretreated group than in saline or isotype control-pretreated groups (**Figures 2E–G**), indicating a general ameliorating effect of anti-IL-1R7 on inflammation *in vivo* in the *P. acnes*/LPS-challenged mice. No significant changes were observed in the temperature or blood white blood cell (WBC) counts among the anti-IL-1R7- or saline- or isotype control- pretreated groups (**Supplementary Figures 4A–E**). No difference was detected in plasma IL-18 levels among the three pretreated groups as well (**Supplementary Figures 4F**).

### Anti-mIL-1R7 protects mice from *P. acnes* and LPS- induced liver injury and inflammation

**Figure 3:**
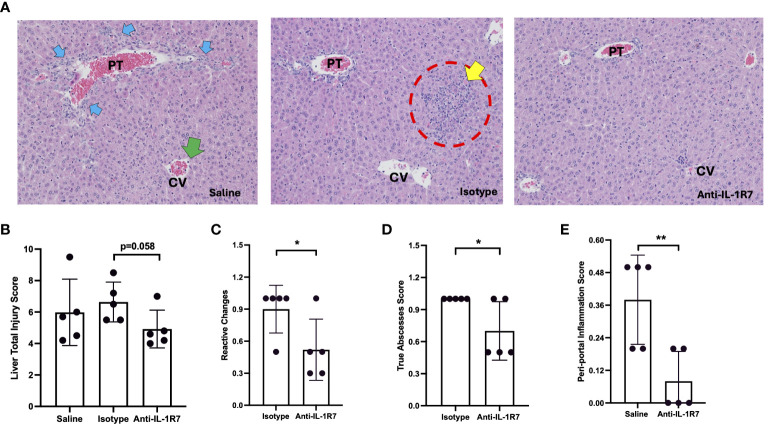
Anti-IL-1R7 pretreatment protects mice from *P. acnes/*LPS-induced liver injury and inflammation. **(A)** Representative images of the H&E-stained mouse liver tissue slices. The mice were challenged with *P. acnes*/LPS in the presence of saline (saline group), or anti-mIL-1R7 (anti-IL-1R7 group) or its isotype control (isotype group) for pretreatment. Yellow arrows: apoptotic cells; Blue arrows: ductal reaction/hyperplasia; Red circle: larger inflammatory cell foci; Green arrows: PMNs in central vein. **(B-E)** Results of liver injury assessment. Total injury score **(B)**, score of reactive changes **(C)**, score of total abscesses **(D)** and score of peri-portal inflammation **(E)** of liver injury and inflammation assessed from the H&E-stained liver tissue slices. Mean ± SD of the liver injury scores of the mice. N=5 for all conditions. ∗∗p < 0.01, and ∗p < 0.05 for comparisons as indicated.

In addition to the ALT and MIP2 measurements, we further investigated the liver injury and inflammation of the *P. acnes*/LPS-challenged mice in more detail. H&E-stained liver slices from the mice above were evaluated blindly using an established score criteria (36). As shown in **Figure 3A**, anti-IL-1R7 pretreatment protected mice from *P. acnes*/LPS-induced liver tissue injury. The anti-IL-1R7 pretreated group showed less total liver injury score than the isotype control-pretreated group, with less reactive changes and reduced true abscess development (**Figures 3B–D**). Mice pretreated with anti-IL-1R7 also presented restrained peri-portal inflammation (**Figure 3E**). In addition, reduced cell injury and migrating polymorphonuclear leukocytes (PMNs) were observed in the anti-IL-1R7-pretreated mice than isotype control- or saline- pretreated mice, although these differences did not achieve statistical significance (**Supplementary Figures 5A, B**).

**Figure 4:**
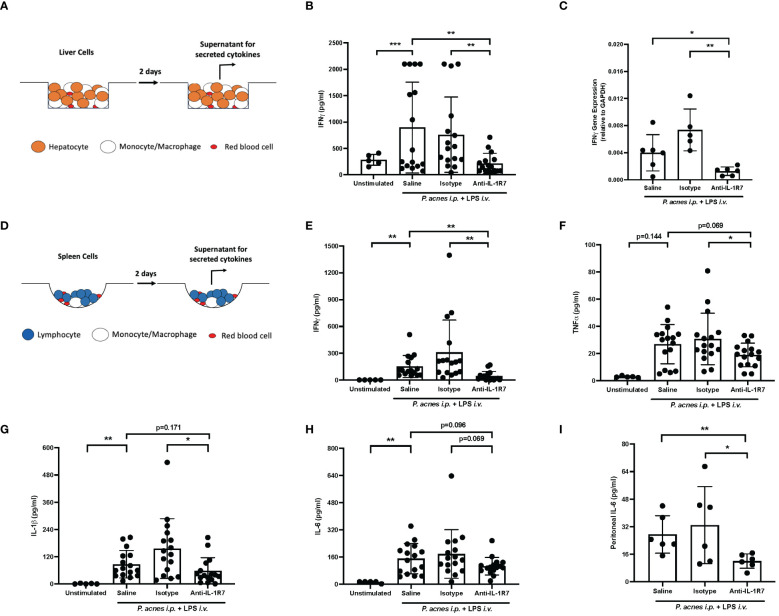
Anti-IL-1R7 suppressed *P. acnes*/LPS-induced liver and spleen cell inflammation. **(A, B)** IFNγ secretion in the *ex vivo* mouse liver cell cultures as depicted in **(A)**. Primary liver cells from the indicated mouse groups were cultured for two days before the supernatant were collected for cytokine measurement. **(C)** *P. acnes*/LPS-induced liver IFNγ mRNA synthesis in mice pretreated with anti-IL-1R7 or its isotype control or saline. Mean ± SD of *P. acnes*/LPS-induced IFNγ steady-state mRNA synthesis in fresh liver tissue samples. **(D-H)** Assessment of *P. acnes*/LPS-induced spleen cell inflammation in the mice. Primary splenocytes from the indicated mouse groups were cultured as described in **(D)**. IFNγ **(E)**, TNFα **(F)**, IL-1β **(G)** and IL-6 **(H)** levels in the supernatant. Mean ± SD of the cytokine levels were shown. **(I)** IL-6 level in peritoneal fluid lavages from mouse groups as indicated. Mean ± SD of *P. acnes*/LPS challenge-induced IL-6 in the peritoneal fluid lavage. N ranges between 5 to 16. ∗∗∗p < 0.001, ∗∗p < 0.01, and ∗p < 0.05 for comparisons as indicated.

Moreover, we assessed liver cell inflammation using *ex vivo* cell cultures (**Figure 4A**). Consistently, primary liver cells collected from anti-IL-1R7-pretreated *P. acnes*/LPS-challenged mice secrete significantly less IFNγ than liver cells collected from either saline- or isotype control- pretreated mice (**Figure 4B**). Besides IFNγ protein expression, anti-IL-1R7 pretreatment also downregulated liver tissue IFNγ gene expression (**Figure 4C**). No significant difference was found in liver WBC counts among the pretreated groups (**Supplementary Figures 4G–J**).

### Anti-mIL-1R7 suppressed *P. acnes* and LPS-induced mouse spleen cell inflammation

**Figure 5:**
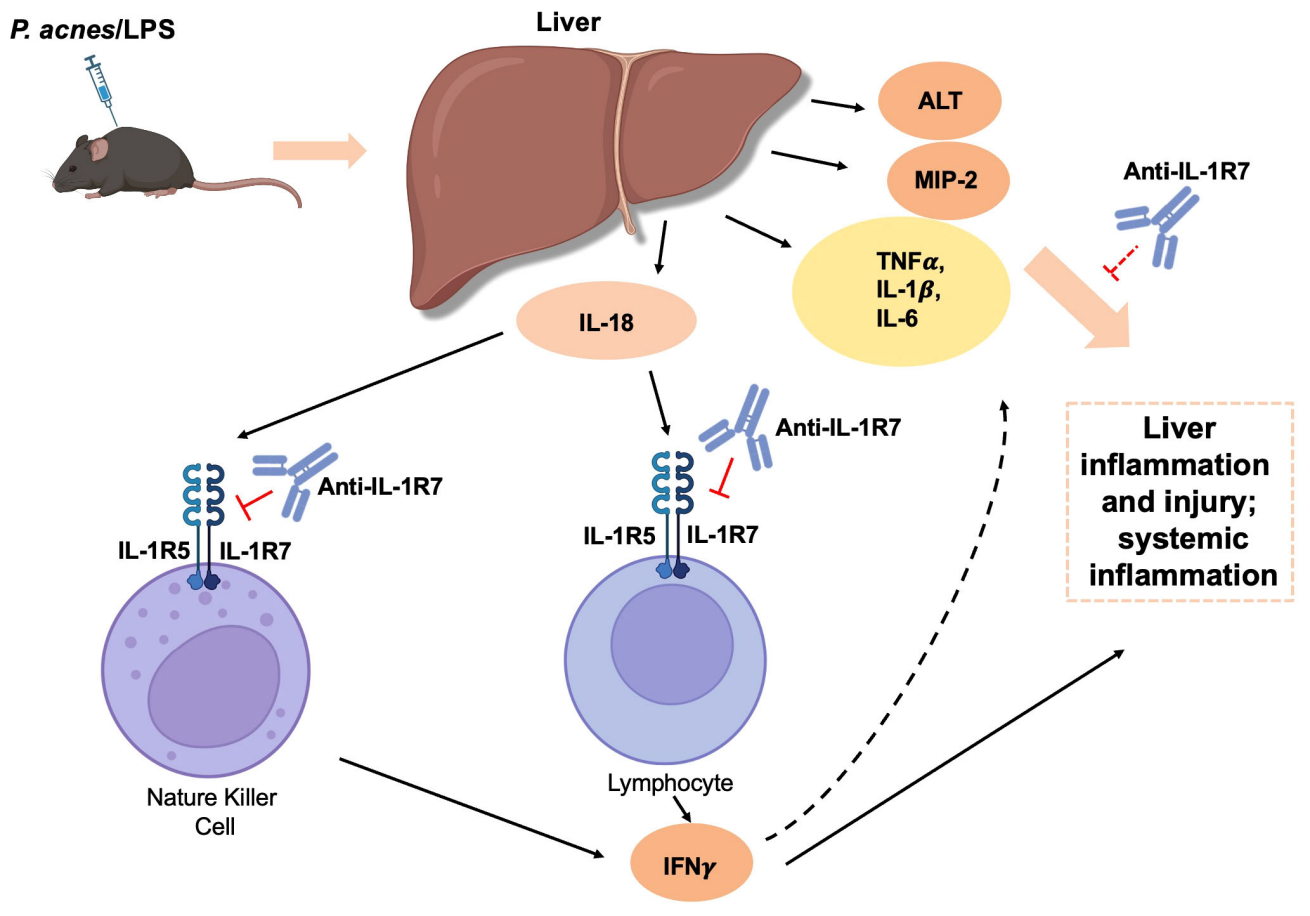
A schematic diagram summarizing the regulation and protective mechanisms of anti-IL-1R7 on the *P. acnes*/LPS-induced liver injury and systemic inflammation. Intraperitoneal injection of *P. acnes*/LPS induces IL-18-mediated liver injury and systemic inflammation in mice. Anti-IL-1R7 suppresses IL-18-induced IFNγ and other cytokine production such as TNFα, IL-1β and IL-6 by blocking the interaction of IL-18 with its receptors IL-1R5/IL-1R7. This subsequently dampens liver inflammation and injury, and protects mice from systemic inflammation. The diagram was created with BioRender contents.

Considering the important role of IL-18 on lymphocyte activation (37), we further examined the effect of anti-IL-1R7 pretreatment on spleen cell inflammation in mice challenged with *P. acnes*/LPS. Similar to the *ex vivo* liver cell culture above, primary spleen cells from either anti-IL-1R7- or saline- or isotype control- pretreated mice were collected for culture (**Figure 4D**). As presented in **Figure 4E**, spleen cells from anti-IL-1R7-pretreated mice released substantially less IFNγ than spleen cells from saline- or isotype control- pretreated mice. In addition, cells from anti-IL-1R7-pretreated mice also secrete less TNFα and IL-1β than cells from isotype control-pretreated mice (**Figures 4F, G**). A reduction in IL-6 production was also observed in the same cells but the results did not reach statistical significance (**Figure 4H**). Similarly, less IL-6 was detected in peritoneal fluid lavage from anti-IL-1R7-pretreated mice than saline- or isotype control- pretreated mice (**Figure 4I**). No significant difference was observed in spleen or peritoneal total WBC counts among the groups (**Supplementary Figures 4K, L**). As a summary, our findings from the *P. acnes*/LPS model are depicted in **Figure 5**.

### Anti-mIL-1R7 treatment reduces LPS-induced lung neutrophilia and protects mice from acute lung injury (ALI)

**Figure 6:**
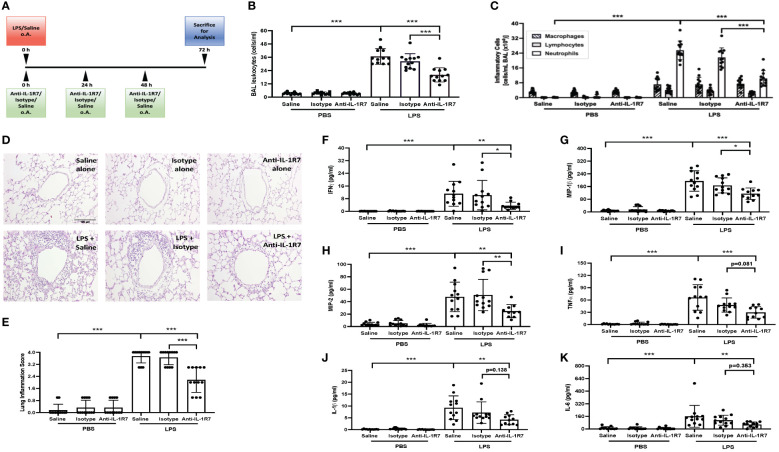
Anti-IL-1R7 protects mice from LPS-induced lung inflammation. **(A)** Treatment protocol for the LPS-induced lung inflammation model. **(B, C)** Total leukocytes **(B)** and the different inflammatory cell counts **(C)** in the BAL fluid collected from mice challenged with or without intranasal LPS, in the presence of anti-IL-1R7 or its isotype control or saline for treatments. **(D)** Representative images of the H&E-stained lung tissue slices. **(E)** Statistic analysis of the lung inflammation score of the H&E-stained lung tissue slices. **(F)** IFNγ, **(G)** MIP-1β, **(H)** MIP-2, **(I)** TNFα, **(J)** IL-1β and **(K)** IL-6 levels in the BAL fluids. Mean ± SD of the cytokine levels were shown. N ranges between 7 to 12. ∗∗∗p < 0.001, ∗∗p < 0.01, and ∗p < 0.05, for comparisons as indicated.

**Figure 7:**
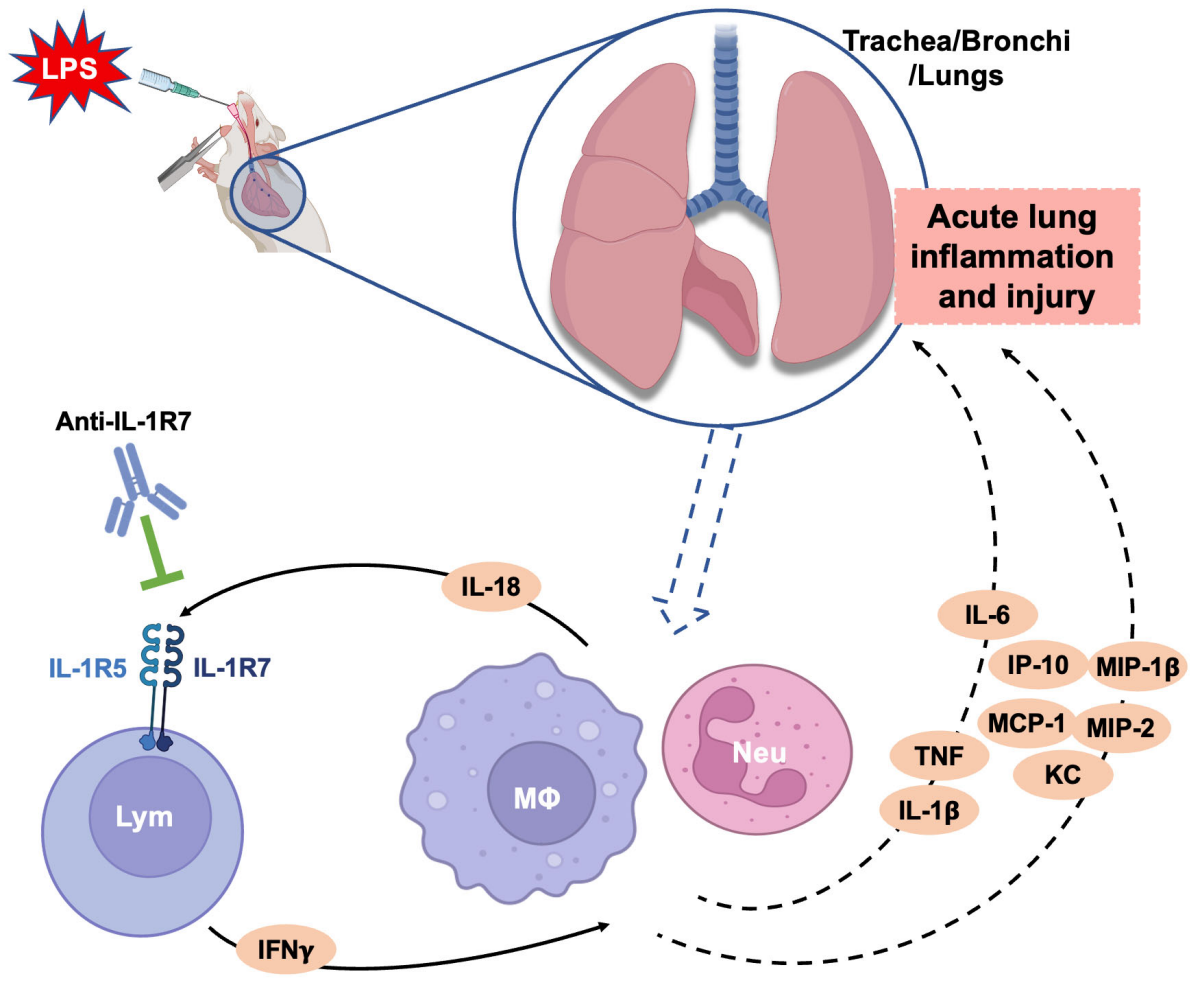
A schematic diagram summarizing the ameliorating effects of anti-IL-1R7 on the LPS-induced acute lung injury. Intranasal LPS challenge in mice induces strong infiltration of neutrophils, macrophages and lymphocytes into the lungs. The infiltrated inflammatory cells secrete many proinflammatory cytokines and chemokines which lead to acute lung injury. Anti-IL-1R7 blocks IL-18-induced IFNγ by inhibiting the interaction of IL-18 with its receptors IL-1R5/IL-1R7, and downregulates the secretion of other associated cytokines and chemokines including TNFα, IL-1β and IL-6, MIP-1β, MIP-2, IP-10 and MCP-1. Lym, lymphocyte; MΦ, macrophage; Neu, neutrophil. The diagram was created with BioRender contents.

Elevated IL-18 and IFNγ levels were found in COVID-19 patients recently and their levels are known to be correlated to various lung diseases including ALI and acute respiratory distress syndrome (ARDS) (8, 38–40). Therefore, in addition to the *P. acnes*/LPS-induced liver injury and systemic inflammation model, we evaluated the influence of IL-18/IL-1R7 blockage by anti-IL-1R7 in a standardized mouse model of LPS-induced ALI (**Figure 6A**). Intranasal application of LPS induced strong infiltration of leukocytes and especially neutrophils into the lungs associated with increased secretion of proinflammatory cytokines and chemokines (**Figure 6**, **Supplementary Figure 6**). Anti-IL-1R7-treatment significantly lowered total leukocytes and neutrophil counts in the bronchoalveolar lavage (BAL) fluid and suppressed LPS-induced lung inflammation as compared to either saline- or isotype control- treated groups (**Figures 6B–E**). Alike in the *P. acnes*/LPS model, IFNγ production was significantly reduced by anti-IL-1R7-treatment (**Figure 6F**). In addition, BAL levels of proinflammatory mediators, especially MIP-1β, MIP-2, TNFα, IL-1β and IL-6 were also lower after anti-IL-1R7-treatment (**Figures 6G–K**). A summary of our findings with the potential functional mechanism in the ALI model are summarized in **Figure 7**.

## Discussion

In conclusion, our data from the present study showed that anti-mIL-1R7, an antibody against mIL-1R7 (the co-receptor of IL-18), significantly inhibited IL-12/IL-18- or LPS- stimulated IFNγ production in mouse spleen cells and peritoneal cells, protected mice from *P. acnes*/LPS- induced liver injury and systemic inflammation, and prevented mice from LPS-induced acute lung injury and inflammation. To our knowledge, this is the first time an anti-IL-1R7 antibody has been examined *in vivo* for suppression of hyperinflammation.

Inflammation is the normal response to infection and injury. However, when allowed to continue unchecked, inflammation can result in severe diseases with high mortality including MAS and MAS-associated COVID-19. IL-18 is known to play an important role in the hyperinflammation and disease development (8, 10, 41–46). Known as secondary hemophagocytic lymphohistocytosis (sHLH), MAS is characterized by a lethal hyper-inflammatory state with liver dysfunction, pancytopenia, increased D-dimer and ferritin, and coagulopathy (5). A severe IL-18/IL-18BP imbalance was observed in MAS patients where the plasma concentrations of IL-18 were 20–30 times higher than in patients with rheumatic arthritis (44, 47–50). Markedly increased plasma IL-18 levels are also present in patients with systemic juvenile idiopathic arthritis (sJIA) or systemic inflammatory adult-onset Still’s disease (AOSD), which are at high risk of developing life-threatening MAS (43, 48, 50–52). Anakinra, a natural antagonist for the IL-1 Receptor, is effective in treating patients with sJIA or AOSD who develop MAS (5, 53, 54), with a mechanism involving a reduction in the processing of precursor IL-18 into an active cytokine (55). IL-18BP, the natural inhibitor of IL-18, also demonstrated beneficiary outcomes with early signs of clinical and laboratory marker efficacy in treating patients with refractory AOSD and sJIA (56, 57). Similar to MAS, patients with a gain-of-function mutation in *NLRC4* (58) or deficiency in X-linked inhibitor of apoptosis (XIAP) (41) also experience a life-threatening hyper-inflammatory state with high levels of free IL-18. Treatment of these patients with IL-18BP alleviates the inflammatory state (5). In children with the *NLRC4* mutation, IL-18BP also ameliorated the severe life-threatening colitis in clinical studies (58). In parallel, blocking of IL-12, IL-18 or its downstream effector IFNγ can down-regulate the severity of experimental IBD in mice (59–61). Neutralization of IL-18 with IL-18BP or anti-IL-18 antibodies demonstrated efficacy in dextran sodium sulfate (DSS)- or trinitrobenzoic sulphonic acid (TNSB)- induced models of IBD, and reduces intestinal IFNγ and TNFα, further proving the concept of IL-18 as a pivotal mediator in experimental colitis (60, 62, 63). Altogether, these findings suggest that IL-18 neutralization can contribute to the resolution of the hyper-inflammatory state in disease.

As discussed earlier in detail (6), in any IL-18-related pathological condition, the outcome of blocking IL-18 correlates with the concentration of free, active IL-18, the surface level of IL-1R5, the presence of IL-1R7 and IL-18BP (1). In diseases with hyperinflammation, such as MAS, large quantities of free IL-18 are produced to bind IL-1R5 and less IL-1R5 becomes available for the anti-inflammatory IL-37. On the other hand, if the concentration of IL-18BP increases and exceeds the need to bind IL-18, IL-37 can bind to the excess IL-18BP and is not available for promoting its anti-inflammatory portfolio (14, 21, 32, 64). This concept fits well with a recent finding from a Dutch study where 300 patients at high risk for a cardiovascular event had high levels of IL-18BP (64). In that study, biomarkers of risk such as CRP correlated with the level of IL-18BP. The concept also explains the association of low IL-37 levels with inflammatory diseases and the diminished anti-inflammatory properties of IL-18BP at high doses (16–19). In addition, we observed in a parallel study that high doses of IL-18BP treatment increase inflammatory eosinophil infiltration into the lungs of ovalbumin (OVA)- sensitized mice, indicating a potential risk of high-dose IL-18BP in asthma development (**Supplementary Figure 7**). In contrast, as the sole accessory chain for IL-1R5 (24), IL-1R7 is essential for the recruitment and activation of IRAK for IL-18-induced signaling and function (65–68). Unlike IL-18BP, which directly binds to IL-18, anti-IL-1R7 targets the IL-18 co-receptor IL-1R7. Although recombinant IL-18BP is much more potent in inhibiting IL-18-induced IFNγ (**Figure 1**, **Supplementary Figure 1**) (6), anti-IL-1R7 can be a better strategy. Recombinant IL-18BP is so potent that even at 1 μg/mL, it completely abolished IL-18-induced IFNγ in cell culture. However, considering the important role of IFNγ in host defense against opportunistic infections (most importantly *Mycobacterium tuberculosis*) (69–71), a basal level is needed to maintain a healthy balance. Anti-IL-1R7 is ideal with which IL-18-induced IFNγ level is dampened but not completely abolished (**Figure 1**) (6). In addition, our data proved that anti-IL-1R7 does not inhibit the anti-inflammatory function of IL-37. This is another advantage we seek in anti-IL-1R7. It is noteworthy that our data showed that both anti-IL-1R7 and overexpression of IL-37 independently reduced the production of IFNγ induced by LPS (70% reduction by anti-IL-1R7 and 65% reduction by overexpression of IL-37). The combined use of anti-IL-1R7 and IL-37 overexpression further decreased IFNγ production (~ 90% reduction), indicating a synergistic effect. This synergistic effect could result from the combined inhibitory effects from anti-IL-1R7 and IL-37, either independently or dependently. This mechanism is worthy of further investigation.

In this study, we selected *P. acnes*/LPS-induced liver injury and systemic hyperinflammation as the primary model to evaluate the function of anti-IL-1R7 *in vivo* as it is the classic model where mIL-18 was originally discovered as the IFNγ-inducing factor and a robust production of IFNγ was observed (2, 33, 72). In line with the original discovery, we detected large amounts of IFNγ in the plasma, as well as in the *ex vivo* liver and spleen cell cultures from mice challenged with *P. acnes*/LPS. High levels of other proinflammatory cytokines such as TNFα, IL-1β and IL-6 were also observed in the plasma, indicating a systemic hyperinflammation in this model. We also detected significant liver injury and inflammation in the *P. acnes*/LPS-challenged mice (liver tissue pathology and elevated plasma ALT and MIP2). Anti-IL-1R7 pretreatment remarkedly suppressed plasma IFNγ and other inflammatory cytokines, confirming the important role of IL-18 in mediating the systemic inflammation and the efficacy of anti-IL-1R7 strategy in suppressing IL-18-induced hyperinflammation. Anti-IL-1R7 pretreatment also reduced IFNγ production from liver and spleen cells and ameliorated *P. acnes*/LPS-induced liver damage and spleen cell inflammation, further demonstrating the protective properties of anti-IL-1R7 in IL-18-mediated local and systemic inflammation (**Figure 5**). Our study utilized a mixture of cells from the liver or spleen because different cells are involved in the IL-18-IFNγ-inflammation cascade (5). IL-18 is secreted by macrophages or dendritic cells (IL-18 producers) in response to LPS or other inflammatory insults (5, 8). The secreted IL-18 can then activate IL-18 effector cells, including T lymphocytes and natural killer (NK) cells, which express both the IL-18 receptor and co-receptor (IL-1R5 and IL-1R7). The IL-18 effector cells thus produce IFNγ, which can further stimulate and interact with other immune cells such as macrophages and neutrophils to amplify the inflammatory cascade (**Figures 5**, **7**). Anti-IL-1R7, which targets IL-1R7 on the cell surface of IL-18 effector cells, inhibits IL-18 activation in the cells to impede the subsequent IL-18-IFNγ-inflammation cascade. In the *P. acnes*/LPS model, *P. acnes* was first applied to prime the immune system leading to the formation of granuloma consisting of infiltrated mononuclear cells/Kupffer cells in hepatic lobules (73) and the subsequent LPS injection activates the macrophages to initiate a series of inflammatory response leading to liver injury and a hyperinflammatory condition. In this model, the mice were only challenged with LPS for 3 hours (h). Some report indicated that prolonged *P. acnes/*LPS challenge may induce mouse mortality (74). In a preliminary study, we monitored the *P. acnes*/LPS-challenged mice for 48 h. However, no significant lethality was found, although the mice were very sick during the first 24 h after LPS injection. This may be explained by the difference in *P. acnes*/LPS tolerance between the mouse strains used. However, it is noteworthy that in comparison to the isotype control-pretreated mice, the mice pretreated with anti-IL-1R7 in general showed less weight loss and temperature drop in response to LPS (**Supplementary Figure 8**), suggesting a protective effect by anti-IL-1R7 pretreatment in general.

As an addition to the *P. acnes*/LPS- induced liver injury and systemic hyperinflammation, we explored the function of anti-IL-1R7 in LPS-induced local inflammation using the mouse model of ALI. The effects of anti-IL-1R7 on leukocyte migration (especially neutrophils) in response to LPS challenge, as well as the suppression on lung inflammation suggest an important role of IL-18 in respiratory inflammatory diseases. This is in line with the high levels of IL-18 found to be associated with disease severity and poor clinical outcome in COVID-19 patients (10, 12, 38, 75, 76). The COVID-19 pandemic has brought attention to a virally induced hyperinflammatory lung injury, sometimes evolving to ARDS, the most severe form of acute lung injury, that is characterized by a cytokine storm syndrome, multiple-organ failure and in most cases leads to death (77–81). This finding also mirrors what has been observed in MAS (82–84). In fact, MAS is present in patients with severe COVID-19 disease status and is highlighted as a parallel with the life-threatening COVID-19 infection because of the genetic, clinical, histopathological, cytological and immunological similarities (11, 45, 85–90). A significantly higher serum IL-18 level was observed in the COVID-19 patients with MAS than in the patients without MAS (11, 85) and the elevated IL-18 level is associated with disease severity and poor clinical outcome in COVID-19 patients (12, 75). In addition to the high levels of IL-18, IL-1R7 is also found to be highly expressed in cell-to-cell communication among immune cells in COVID-19 patients and an elevated IFNγ was also observed (38, 75, 76, 91, 92). Moreover, in another respiratory disease SARS, caused by SARS-CoV-1 virus, IL-18 concentration was also elevated in the patients than those in healthy subjects, and higher levels were observed in nonsurvivors than survivors (93, 94). Similarly, IL-18 was found to be involved in an IFNγ-related cytokine storm in the patients (93). It is important to note that increased IL-18 levels were also found to be associated with severe forms of asthma and chronic obstructive pulmonary disease (COPD) and ARDS (8). Elevated IL-18 concentrations were observed in the serum and lungs of patients with ARDS and correlated with severity score and death (39, 95). Altogether, all the pieces of evidence demonstrate an important role of IL-18 in severe forms of respiratory diseases and the pertinency of anti-IL-1R7 in treating IL-18-mediated respiratory inflammation. However, the exact mechanism remains unknown and is worthy of further study.

In summary, results from our studies further validated IL-1R7 as a potential therapeutic target and set the stage for a full characterization of the potential of anti-IL-1R7 in clinical studies of IL-18-mediated diseases such as MAS, COVID-19, IBD and rheumatic diseases (5, 8). Patients carrying the NLRC4 mutation with life-threatening enterocolitis could also benefit from such an antibody specific to IL-18 inhibition (58). Further investigation on the application of anti-IL-1R7 will not only provide new mechanistic insights into the function of IL-18 in disease, but also will likely identify novel therapeutic targets for treating IL-18-mediated diseases.

## Material and methods

### Antibodies and reagents

The anti-mouse IL-1R7 antibody was generated by immunization of New Zealand white rabbits (Charles River Laboratories, Wilmington, MA) with mouse recombinant IL-1R7 protein. Anti-mouse IL-1R7 antibody and non-binding isotype control antibody were produced as mIgG2a-LALA isotype in HEK293-FreeStyle cells from Thermo Fisher Scientific (Waltham, MA, # R79007) and purified from the supernatant using protein-A affinity chromatography followed by size exclusion chromatography (MAB Discovery GmbH, Neuried, Germany). The antibodies have an incorporated double substitution, LALA that significantly reduces binding to FcγRs to avoid Fc-mediated effector functions (30, 31). The antibodies were then dissolved in the buffer with 20 mM Histidine, 140 mM NaCl at pH 6, divided into aliquots and stored at -80 °C before use. Lipopolysaccharide (LPS) *Escherichia coli* (055:B5) was purchased from Sigma-Aldrich (St Louis, MO, # L2880–10MG) unless otherwise specified. *P. acnes* LyfoDisk (also known as *P.acnes* ATCC11827; formerly designated “Corynebacterium parvum” was purchased from Microbiologics (Cloud, MN, # 23–016-648). Mouse IL-18 and IL-12 were from Bio-Techne (Minneapolis, MN, # 9139-IL-010 and # 419-ML-010). Clinical grade recombinant human IL-18BP was a gift provided by Serono pharmaceutical research institute (SPRI, Geneva, CH). For cytokine measurements related to the liver injury model, the corresponding ELISA DuoSet kits for mouse cytokines including IL-1β, TNFα, IL-6, IFNγ, IL-18 and MIP-2 were from Bio-Techne (Minneapolis, MN, # DY401, # DY410, # DY406, # DY485, # DY7625–05, # DY452). ALT measurement kit was purchased from MedTest Dx (Canton, MI, USA, # A7526–150). For cytokine measurements associated with the ALI model, the levels of murine IL-1β, IL-6, IL12p70, IL-18, IFNα, IFNγ, IP-10, MCP-1, MIP-1β, MIP-2 and TNFα in BAL samples were assessed using MSD U-Plex Assays (Meso Scale Diagnostics, Rockville, MD, USA) according to the manufacturer´s guidelines.

### Generation of thioglycollate-elicited mouse peritoneal cells

The animal protocols were approved by University of Colorado Animal Care and Use Committee unless otherwise specified. Thioglycollate-elicited peritoneal cells from age-matched WT and IL-37Tg male mice were generated as previously described (96). 1 mL of 3.7% Brewer’s autoclaved thioglycollate medium was instilled intraperitoneally in WT or IL-37Tg mice. On the 4th day after the instillation of the thioglycollate, the mice were euthanized. 10 mL of RPMI was introduced into the cavity and peritoneal fluid lavage was collected for cell counts and peritoneal cell culture. The peritoneal cells were seeded at 1x10^6/mL in RPMI culture medium (Corning, Corning, NY, # 10–040-CV) on 24-well plate in the presence of 10% FCS (Corning, # 35–011-CV) and 1% penicillin/streptomycin (Corning, # 30–002-CI). The cells were pretreated with or without 5 μg/mL anti-mIL-1R7 for 30 minutes before they were stimulated with 1 μg/mL LPS for 24 h at 37°C. The supernatants were collected for cytokine measurement.

### Generation of heat-inactivated *P. acnes*

Pellets of the *P. acnes* LyfoDisk were resuspended in sterile PBS at the concentration of 50 mg/mL. The *P. acnes* were heat-inactivated at 60°C in water bath for 1 h as described earlier (33, 72).

### Mouse model of *P. acnes* and LPS-induced tissue and systemic inflammation

Gender- and age- matched male and female mice (8-week-old) were injected intraperitoneally (i.p.) with 0.2 mL of PBS containing 10 mg of heat-killed *P. acnes* on Day 1. In parallel, in another set of gender- and age- matched mice, 0.2 mL PBS alone was injected as vehicle control. On Day 7, the *P. acnes*-injected mice were treated i.p. with either 0.2 mL saline, or 0.2 mL of 20 mg/kg isotype control antibodies or anti-mIL-1R7 antibodies in saline. The vehicle control mice were injected with 0.2 mL saline. On Day 8, the mice were treated again with the same solution as on Day 7 (either 0.2 mL saline, or 0.2 mL of 20 mg/kg isotype control antibodies or anti-mouse IL-1R7 antibodies in saline). In 30 minutes, the *P. acnes*-primed mice were further challenged intravenously (*i.v.)* with 1 μg of LPS in 0.2 mL of PBS, whereas the vehicle control mice were challenged *i.v* with 0.2 mL of PBS alone. The mice were euthanized 3 h after LPS injection. Blood was collected for plasma cytokine and liver enzyme measurement. Peritoneal fluid lavage was collected for cell counts and cytokine measurement. Liver cells and splenocytes were collected for *ex vivo* cell culture. Part of the liver were collected and fast frozen in liquid nitrogen and stored at -80°C for later use such as mRNA analysis.

### Assessment of mouse liver histopathology

For liver injury scoring, the mouse livers were collected and fixed in 10% formalin. The livers were then embedded in paraffin, sectioned, and stained with H&E (University of Colorado Denver Morphology and Phenotypic Core). Histological examination was performed and evaluated blindly as previously described using an established score criteria (36, 97). The entire cross section of liver was analyzed from each mouse for liver injury. Images were captured on an Olympus BX51 microscope equipped with a 4 megapixel Macrofire digital camera (Optronics) using the PictureFrame Application 2.3 (Optronics) (36).

### *Ex vivo* mouse liver cell cultures

Liver was removed from each mouse challenged with *P. acnes* and LPS as above. The liver tissue was weighed and cut into small pieces in saline with sterile utensils. The liver pieces were broken down gently using a syringe plunger tip and the cell suspensions were passed through a 100 μm cell strainer. The cells were gently washed twice with saline at 1,000 rpm for 5 minutes and resuspended in RPMI culture medium at 0.2 g/mL. 1 mL of the liver cell suspensions were seeded per well on 12-well cell culture plates. The cells were cultured for 48 h before the supernatant and cell lysates were collected. Supernatants were collected by centrifugation at 400 x g for 5 minutes and stored at -80°C for cytokine analysis. Cells remaining in the wells were lysed in 200 μl 0.5% triton-X in water and stored at -80°C for later use such as total protein quantification.

### Mouse liver gene expression

Part of the liver was collected from each mouse and homogenized for RNA purification using TRIzol reagent (Invitrogen, Carlsbad, CA, # 15596018). The RNA was reverse transcribed using High Capacity cDNA Reverse Transcription Kit (Applied Biosystems, Waltham, MA, # 4368814) and quantitatively measured for gene expressions using PowerSYBR Green PCR Master Mix (Applied Biosystems, # 4367659). Mouse IFNγ mRNA synthesis was measured and mouse GAPDH was used as the internal control. The forward primer for mouse GAPDH was: 5’ TTCAACAGCAACTCCCACTCTTCCA 3’. The reverse primer for mouse GAPDH was: 5’ ACCCTGTTGCTGTAGCCGTATTCA 3’. The forward primer for mouse IFNγ was: 5’ CAGCAACAGCAAGGCGAAAAAGG. The reverse primer for mouse IFNγ was: 5’ TTTCCGCTTCCTGAGGCTGGAT 3. The relative ratio of the mRNA from IFNγ gene to internal control (GAPDH) was calculated as: 1/2^Δ^ *^Ct^* (cytokine gene minus the internal control gene).

### Mouse spleen cell culture

The spleen cell suspensions were obtained as described (96) and passed through a 70 μm sterile cell strainer. Cells were washed with RPMI and resuspended in RPMI supplemented with 1% P/S and 10% FCS at 1x10^7 cells/mL. For *in vitro* assessment of the effects of anti**-**IL-1R7, cells were seeded on 96-well round-bottom plates with or without the pretreatment of anti**-**IL-1R7, or its isotype control or IL-18BP for at least 30 minutes. The cells were then stimulated with or without IL-12/IL-18 or 1 μg/mL LPS for 24 h in a humidified atmosphere with 5% CO2. 2 ng/mL IL-12 + 20 ng/mL IL-18 and 1 x 10 ^ 6 splenocytes per well were used for antibody clone screening and the dose responses of the antibodies. After the optimal antibody candidate was selected, 1 ng/mL IL-12 + 10 ng/mL IL-18 and 0.5 x 10 ^ 6 splenocytes per well were used for subsequent cell culture studies. For *P. acnes*/LPS mouse model study, spleen was collected from each mouse challenged with *P. acnes*/LPS as above. 1 x 10 ^ 7 cells were seeded per well on 24-well plates for 48 h in a humidified atmosphere with 5% CO2. The supernatant was then collected for cytokine measurement.

### Immobilized ELISA binding of anti-mIL-1R7 to mouse IL-1R7

The assay was performed as previously described (6). Nunc 384-well MaxiSorp plates were coated with recombinant mouse IL-1R7 extracellular domain (mIL-1R7-FC; MAB Discovery GmbH, at a concentration of 0.5 µg/mL in PBS for 60 minutes at room temperature. Plates were washed three times with wash buffer (PBS 0.1% Tween) and blocked with PBS, 2% BSA, 0.05% Tween for 60 minutes at room temperature. After three washes with wash buffer, antibodies were added in ELISA buffer (PBS, 0.5% BSA, 0.05% Tween) at different concentrations and were incubated for 60 min at room temperature. Plates were washed three times with wash buffer, followed by incubation with goat anti-mouse-F(ab)_2,_ peroxidase-linked secondary antibody (Invitrogen, # A24512) at a dilution of 1:5000 in ELISA buffer for 60 minutes at room temperature. Plates were washed six times with wash buffer before TMB substrate solution (Invitrogen, # 501129758; 15 µl/well) was added. After 5 minutes of incubation, stop solution (1M HCl, 15 µl/well) was added and absorbance (450 nm/620 nm) measured using a Tecan M1000 plate reader.

### Cell binding of anti-mIL-1R7 to mouse IL-1R7

Similarly, as described before (6), HEK-293-FreeStyle™ cells were transfected with DNAs encoding full-length mouse IL-1R7 and using 293-Free™ Transfection Reagent (Merck, Kenilworth, NJ, # 72181). 24 h after transfection, cells were seeded in a 96-well round bottom plate at a cell density of 1x10^6^ cells/mL in stain buffer (BD, Franklin Lakes, NJ, # 554656). Anti-mIL-1R7 antibody was added at different concentrations and incubated for 1h in the dark at 4°C. Cells were washed once with 150 µL DPBS and incubated with Alexa Fluor 488-conjugated goat F(ab)2 anti-mouse IgG (H+L) (Jackson ImmunoResearch Laboratories, West Grove, PA; #109–546-003) at a concentration of 0.8 µg/mL in stain buffer. Cells were washed once with 150 µL DPBS and resuspended in 150 µL stain buffer containing 1:500 diluted DRAQ7 solution (Abcam, Cambridge, UK; #ab109202; 0.3 mM). Cells were analyzed using a BD FACSVerse flow cytometer.

### Mouse models of LPS-induced ALI and OVA-induced experimental allergic asthma (EAA)

Female wild-type C57BL/6 mice, aged 6–8 weeks, were housed under specific pathogen-free conditions receiving OVA-free diet and water *ad libitum*. All animal studies were in accordance with the German animal protection law and were approved by the local animal research ethics board (V244–230826/2015 (76–11/21)). ALI was induced by intranasal application of 10 µg LPS (Sigma-Aldrich, # L4391) in 50 µL saline via oropharyngeal aspiration (o.A.). Subsequent intranasal anti-mIL-1R7 antibody (200 µg in 50 µL saline) treatment started 1 h after LPS application and was repeated after 24 h and 48 h. 72 h after LPS application airway hyperresponsiveness was assessed, all animals were sacrificed by cervical dislocation under deep anesthesia and sampling (serum, broncho-alveolar lavage (BAL), lung tissue) was performed. EAA was induced as described previously (98). All animals were sacrificed by cervical dislocation under deep anesthesia. Sampling (serum, BAL, lung tissue) was performed 24 h after the last OVA challenge. Mice challenged with OVA aerosol received either PBS or recombinant IL-18BP o.A. as a treatment. Animals for the negative control group were sham sensitized to PBS and subsequently challenged with OVA aerosol and were treated with PBS o.A. (healthy group).

For lung histology and inflammatory scoring, the mouse lungs were collected and inflated with phosphate buffered 4% PFA (Roth, Karlsruhe, Germany, # P087.3) under constant pressure of 20 cm water column for 20 minutes and fixed overnight with phosphate buffered 4% PFA. The lungs were then embedded in paraffin, sectioned, and stained with H&E (Sigma-Aldrich, # HT110132–1L, 1.09249.1000). The entire cross section of lungs was analyzed from each mouse for lung inflammation. Images were captured on an Olympus BX51 microscope equipped with a DP25 digital camera (Olympus) using the Cell^A (Olympus) and evaluated blindly at 200x magnifications by an experienced pathologist in order to describe the histopathological characteristics. Semiquantitative analysis of the inflammatory process was performed using the following graduation: grade 0 (absent), 1 (discrete), 2 (mild), 3 (moderate) and 4 (intense).

### Bronchoalveolar lavage and differential cell count

Lungs were lavaged with 1 mL ice-cold PBS containing protease inhibitor (Complete; Roche, Basel, Switzerland, # 11697498001) via a tracheal cannula. Cells were counted using a Neubauer counting chamber. Aliquots of 50 µL of lavage fluids were cytospinned (Cytospin™; Thermo Fischer Scientific, # A78300004), stained with Diff-Quick (Cella Vision RAL Diagnostics, Düdingen, Switzerland, 720555–0000), and cells were microscopically differentiated according to morphologic criteria as previously described (99).

### Assessment of airway hyperresponsiveness (AHR)

Airway responsiveness to methacholine (MCh, acetyl-β-methylcholine chloride; Sigma-Aldrich, # A2251–25g) challenge in anesthetized and ventilated mice was invasively assessed 72 h after LPS treatment using FinePointe RC Units (Data Science International, St. Paul, MN, USA) by continuous measurement of airway resistance (RI). Animals were weighed and anesthetized with ketamine (90 mg/kg body weight; cp-pharma) and xylazine (10 mg/kg BW; cp-pharma) and tracheotomized with a cannula. Mechanical ventilation was previously described (100). Measurements were taken at baseline (PBS) and in response to inhalation of increased concentrations of aerosolized methacholine (3.125; 6.25; 12.5; 25; 50; and 100 mg/mL). After assessment of lung function, all animals were sacrificed by cervical dislocation under deep anesthesia.

### Statistical analysis

Significance of differences was evaluated with Student’s 2-tail *t* test or one-way ANOVA. For one-way ANOVA, Tukey’s *post hoc* test was employed in pairwise comparison. Excel 16.85 and GraphPad Prism 10.2.3 were used for data analysis. GraphPad Prism 10.2.3 was used for graph preparation. The mean or mean percent change for each measurement was calculated as indicated in the Figure Legends. The data shown represent the Mean ± SD.

## Data availability statement

The original contributions presented in the study are included in the article/**Supplementary Material**. Further inquiries can be directed to the corresponding authors.

## Ethics statement

The animal study was approved by IACUC, University of Colorado Denver | Anschutz Medical Campus. The study was conducted in accordance with the local legislation and institutional requirements.

## Author contributions

LJ: Formal analysis, Investigation, Methodology, Validation, Writing – review & editing. LL: Formal analysis, Investigation, Methodology, Writing – original draft, Writing – review & editing. WW: Formal analysis, Investigation, Methodology, Writing – review & editing, Validation. KB: Formal analysis, Investigation, Methodology, Validation, Writing – review & editing. TA: Investigation, Methodology, Writing – review & editing. JF: Investigation, Methodology, Writing – review & editing. JA: Investigation, Methodology, Writing – review & editing, Visualization. AD: Investigation, Methodology, Writing – review & editing. TN: Investigation, Methodology, Writing – review & editing. UP: Investigation, Methodology, Writing – review & editing. DP: Investigation, Methodology, Writing – review & editing. DO: Investigation, Methodology, Writing – review & editing, Visualization. SF: Methodology, Writing – review & editing, Conceptualization, Funding acquisition, Project administration, Resources, Supervision. MW: Conceptualization, Methodology, Project administration, Resources, Supervision, Writing – review & editing, Funding acquisition, Data curation. CD: Conceptualization, Data curation, Methodology, Project administration, Resources, Supervision, Writing – review & editing, Funding acquisition. SL: Conceptualization, Data curation, Methodology, Project administration, Resources, Supervision, Writing – review & editing, Funding acquisition, Formal analysis, Investigation, Software, Validation, Visualization, Writing – original draft.
