# Supplementary material for: An antibody to IL-1 receptor 7 protects mice from LPS-induced tissue and systemic inflammation
Source: Front Immunol. 2024 Jun 25;15:1427100. doi: 10.3389/fimmu.2024.1427100 (PMC11231367; doi:10.3389/fimmu.2024.1427100)
Supplement: Supplementary file 1 [file DataSheet_1.pdf]

Supporting Information

An Antibody to IL-1 Receptor 7 Protects Mice from LPS-Induced Tissue and Systemic Inflammation

Liqiong Jiang[1,9,#], Lars P. Lunding[2,3,#], William S. Webber[1], Karsten Beckmann[4,10], Tania Azam[1], Jesper Falkesgaard Højen[1,5], Jesus Amo-Aparicio[1], Alberto Dinarello[1], Tom T. Nguyen[6], Ulrich Pessara[4,7], Daniel Parera[4,7], David J. Orlicky[8], Stephan Fischer[4,7], Michael Wegmann[2,3,*], Charles A. Dinarello[1,*] and Suzhao Li[1,*]

[1]Department of Medicine, University of Colorado Denver Anschutz Medical Campus, Aurora, CO 80045, USA
[2]Division of Lung Immunology, Priority Area of Chronic Lung Diseases, Research Center Borstel-Leibniz Lung Center, Borstel, Germany
[3]Airway Research Center North, Member of the German Center for Lung Research (DZL), Germany
[4]MAB Discovery GmbH, Tassilostrasse 2, 82398 Polling, Germany
[5]Department of Infectious Diseases, Aarhus University Hospital, 8200 Aarhus N, Denmark
[6]Mucosal Inflammation Program and Division of Gastroenterology, Hepatology, and Nutrition, Department of Pediatrics, Children's Hospital Colorado, University of Colorado, Aurora, Colorado.
[7]IcanoMAB GmbH, Tassilostrasse 2, 82398 Polling, Germany
[8]Department of Pathology, University of Colorado Denver Anschutz Medical Campus, Aurora, CO 80045, USA
[9]Present address: Shenzhen Eye Hospital, No.18 Zetian Road, Futian District, Shenzhen, Guangdong, China 518040
[10]Present address: BioNTech SE, Forstenrieder Strasse 8-14, 82061 Neuried, Germany

[#]These authors contributed equally to this work.
*To whom correspondence should be addressed: Charles A. Dinarello, Michael Wegmann, and Suzhao Li, Department of Medicine, University of Colorado Denver Anschutz Medical Campus, Aurora, Colorado 80045, USA

Email: cdinare333@aol.com; mwegmann@fz-borstel.de; suzhao.li@cuanschutz.edu

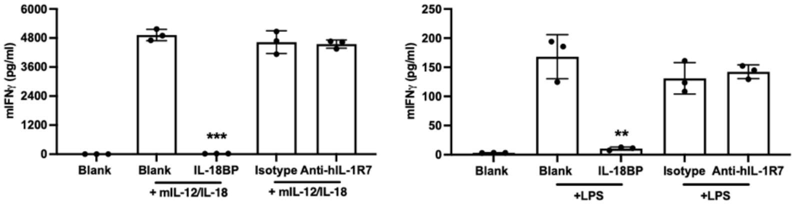

Supplemental Figure 1. Anti-hIL-1R7 monoclonal antibody does not affect mIL-12/IL-18- (Left) or LPS- (Right) stimulated IFNγ production in mouse splenocyte cultures. Mean ± SD of IFNγ production in mouse splenocytes treated with or without anti-hIL-1R7 or its isotype control or IL-18BP for 24 h. N=3. ** $p < 0.01$, *** $p < 0.001$ compared with IL-12/IL-18 or LPS alone-treated cells.

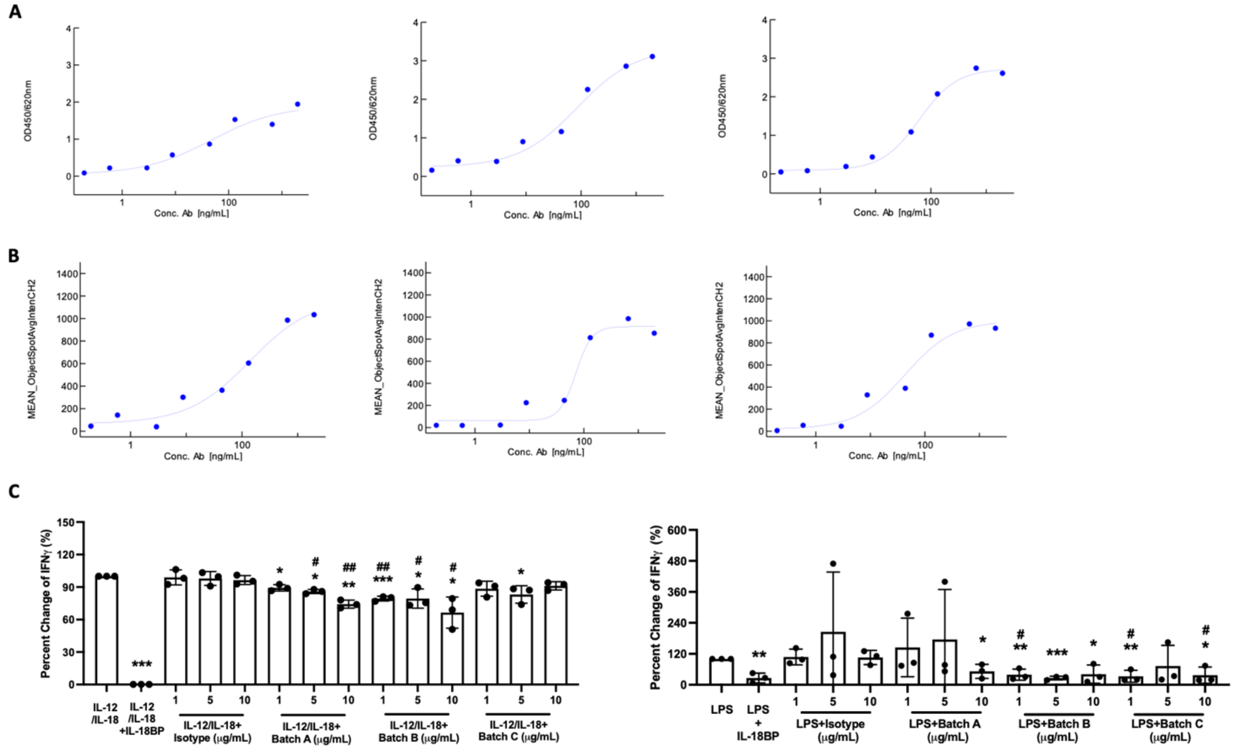

Supplemental Figure 2. Screening of anti-mIL-1R7 antibody monoclonal clones. (A-B) Representative data from three selected anti-mIL-1R7 candidates. Left: Candidate A, Middle: Candidate B and Right: Candidate C. **(A)** dose titration curve for anti-mIL-1R7 binding to immobilized recombinant mouse IL-1R7 protein. ELISA data showing the *in vitro* binding affinity of anti-mIL-1R7 to mouse IL-1R7. **(B)** dose titration curves for anti-mIL-1R7 binding to HEK-293-FreeStyle cells transiently expressing full-length mouse-IL-1R7 encoding DNA. **(C)** Dose effects of the anti-mIL-1R7 candidates on mIL-12/IL-18- or LPS-induced IFNγ production in mouse splenocyte cultures. The cells were pretreated with or without various anti-mIL-1R7 candidate antibodies or the isotype control antibody or 1μg/mL IL-18BP for 30 minutes before the cells were stimulated with or without IL-12/IL-18 or LPS for overnight. N=3. * $p < 0.05$, ** $p < 0.01$, *** $p < 0.001$ compared with IL-12/IL-18 or LPS alone-treated cells. # $p < 0.05$, ## $p < 0.01$ compared with the isotype control-pretreated cells at the same antibody concentration.

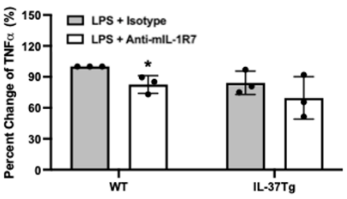

Supplemental Figure 3: Effects of the anti-mIL-1R7 on the anti-inflammatory function of IL-37 on LPS-stimulated TNFα in mouse peritoneal cells. Thioglycolate-elicited peritoneal cells from WT or IL-37-Tg mice were pre-treated with or without anti-mIL-1R7 before LPS treatment (as depicted in Figure 1B). Mean ± SD Percent change of LPS-induced TNFα production (LPS-induced TNFα production in WT cells was set as 100%). N=3 for all conditions. * $p < 0.05$ compared with isotype-pretreated WT cells.

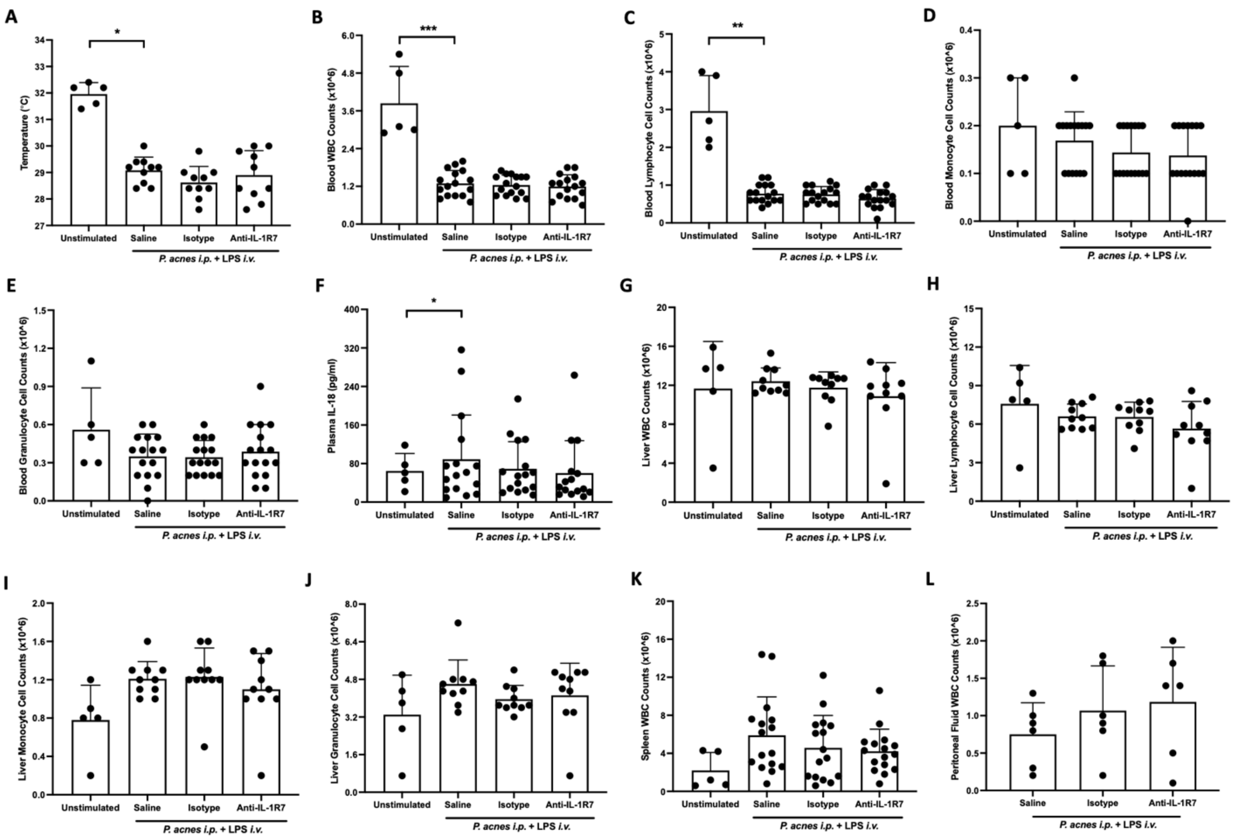

Supplemental Figure 4: Other Parameters measured in the *P. acnes*/LPS-induced tissue and systemic inflammation. **(A)** Temperature measurement. **(B)** Total blood WBC counts. **(C)** Blood lymphocyte cell counts. **(D)** Blood monocyte cell counts. **(E)** Blood granulocyte cell counts. **(F)** Plasma IL-18 levels. **(G)** Total liver WBC counts. **(H)** Liver lymphocyte cell counts. **(I)** Liver monocyte cell counts. **(J)** Liver granulocyte cell counts. **(K)** Total spleen WBC counts. **(L)** Total WBC counts in peritoneal fluid lavage. Mean ± SD of the levels measured in the different groups as indicated. N ranges between 5 to 16. ∗∗∗ $p < 0.001$, ∗∗ $p < 0.01$, and ∗ $p < 0.05$ compared with samples from saline-pretreated and *P. acnes*/LPS-challenged mice (Saline group).

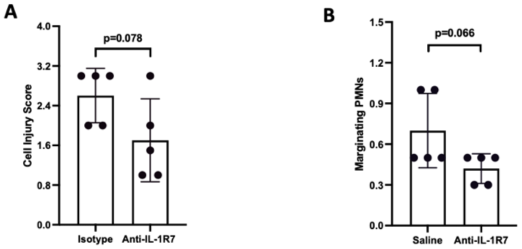

Supplemental Figure 5: Other specific liver injury scores of the *P. acnes/*LPS-challenged mice. (A) Score of cell injury. **(B)** Score of marginating PMNs. Mean ± SD of the specific liver injury scores of the mice challenged with *P. acnes*/LPS in the presence of anti-mIL-1R7 (anti-IL-1R7 group) or its isotype control (isotype group), or saline (saline group) for pretreatment. N=5 for all conditions.

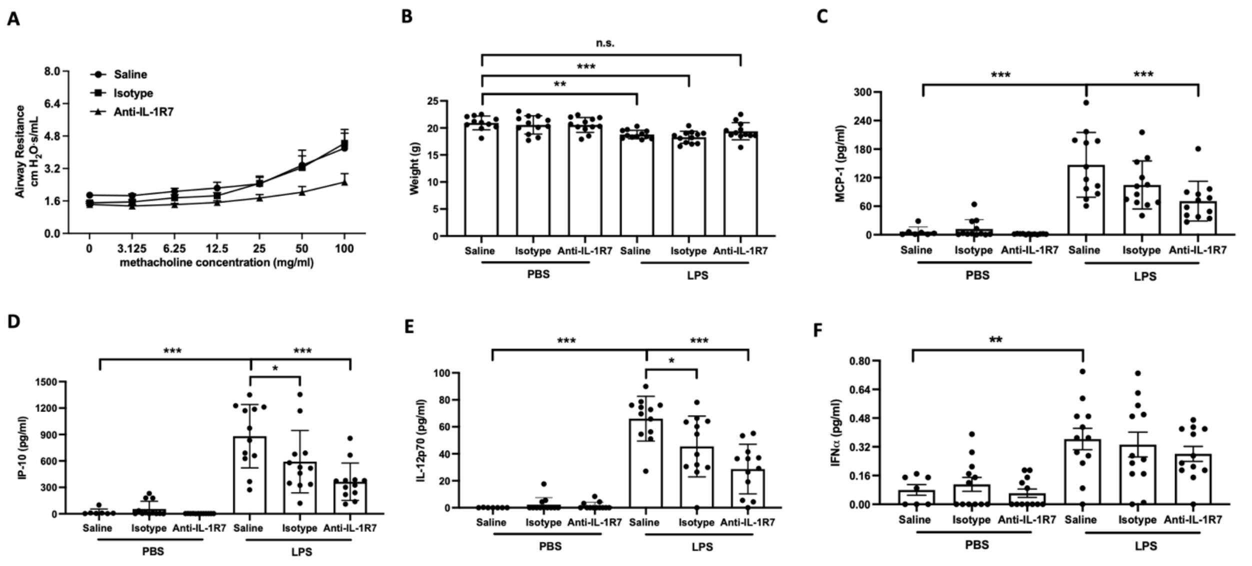

Supplemental Figure 6: Other Parameters measured in the LPS-induced lung injury. (A) Airway resistance measurement in mice treated with saline or isotype control or anti-IL-1R7. **(B)** Body weight measurement. **(C)** MCP-1 level in BAL fluid. **(D)** IP-10 level in BAL fluid. **(E)** IL-12p70 level in BAL fluid. **(F)** IFNα level in BAL fluid. Mean ± SD of measured parameters in anti-mIL-1R7- or its isotype control- or saline- pretreated mice with or without LPS treatment. N ranges between 7 to 12. *** $p < 0.001$, ** $p < 0.01$, and * $p < 0.05$ for comparisons as indicated.

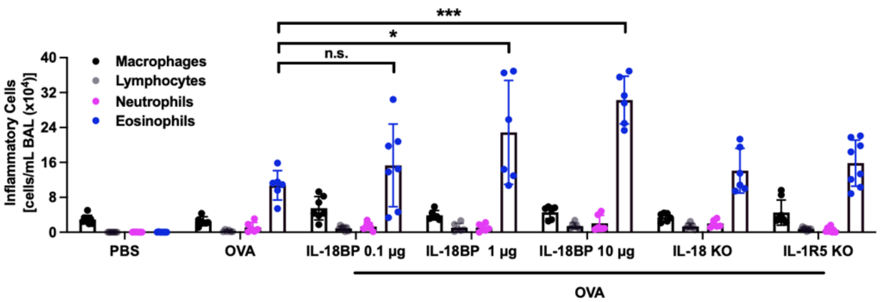

Supplemental Figure 7: Effects of IL-18, IL-1R5 and IL-18BP on OVA inflammation. Cell counts of the different types of leukocytes in BAL fluids collected from mice treated with or without OVA. N ranges between 5 to 7. $*** \ p < 0.001$, and $* \ p < 0.05$ for comparisons as indicated.

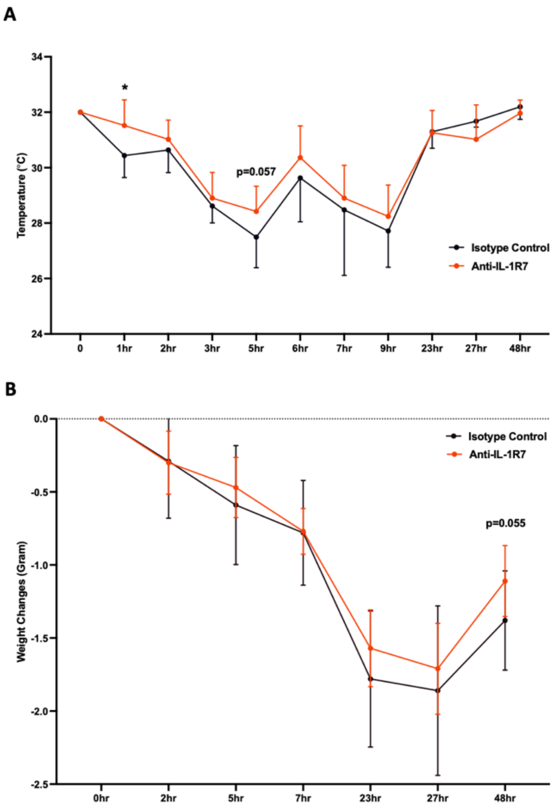

Supplemental Figure 8: **Temperature and weight assessments of mice challenged with *P. acnes*/LPS for a prolonged observation of 48 h for lethality measurement.** The mice were pretreated with either anti-IL-1R7 or its isotype control antibody similarly as depicted in Figure 2A for *P. acnes*/LPS challenge and observed for 48 h for temperature and weight measurements. Mean ± SD of the temperature or body weight changes measured in the mice. N=10 per group. * $p < 0.05$ compared with isotype control-pretreated group.
